# Modulation of CREB in the Dorsal Lateral Geniculate Nucleus of Dark-Reared Mice

**DOI:** 10.1155/2012/426437

**Published:** 2012-01-12

**Authors:** Thomas E. Krahe, Tania A. Seabrook, Ching-Kang J. Chen, Michael A. Fox, William Guido

**Affiliations:** ^1^Department of Anatomy and Neurobiology, Virginia Commonwealth University Medical Center, 1101 E. Marshall Street, Richmond, VA 23298, USA; ^2^Department of Biochemistry and Molecular Biology, Virginia Commonwealth University Medical Center, Richmond, VA 23298, USA

## Abstract

The cAMP-response element-binding protein (CREB) plays an important role in visual cortical plasticity that follows the disruption of sensory activity, as induced by dark rearing (DR). Recent findings indicate that the dorsal lateral geniculate nucleus (dLGN) of thalamus is also sensitive to altered sensory activity. DR disrupts retinogeniculate synaptic strength and pruning in mice, but only when DR starts one week after eye opening (delayed DR, DDR) and not after chronic DR (CDR) from birth. While DR upregulates CREB in visual cortex, whether it also modulates this pathway in dLGN remains unknown. Here we investigate the role of CREB in the dLGN of mice that were CDR or DDR using western blot and immunofluorescence. Similar to findings in visual cortex, CREB is upregulated in dLGN after CDR and DDR. These findings are consistent with the proposal that DR up-regulates the CREB pathway in response to decreased visual drive.

## 1. Introduction

Long-lasting changes in neuronal circuitry are known to require nuclear gene expression and protein synthesis [1]. A critical component is the cAMP-response element (CRE)/CREB transcriptional pathway [2]. CREB is regulated by phosphorylation in response to neuronal activity [3], and its function in long-term plasticity, memory formation, and circuit development has been demonstrated in several neuronal systems [4–7]. In particular, studies in the developing visual cortex reveal a role for CREB in the rearrangement of connections in response to altered sensory input [8–11].

One of the key protocols for examining such experience-dependent plasticity in the visual cortex is raising animals in complete darkness. Dark rearing (DR) has been shown to increase the synaptic strength of excitatory intracortical synapses [12], lower dendritic spine density of pyramidal neurons [13], and enhance long-term potentiation and decrease long-term depression of layer III visual cortical neurons [14]. These changes have been associated with the upregulation of genes subserving synaptic transmission and neural activity [10]. For example, CREB transcription and translation are significantly increased in the visual cortex of mice reared in complete darkness from birth till postnatal day (P) 27, the peak of cortical plasticity in mice [15]. These changes have been attributed to synaptic compensatory mechanisms that are triggered by a reduction in afferent drive brought about by DR [10].

While CREB has been implicated in the refinement of subcortical visual connections, such as the remodeling of retinal projections from the two eyes to the dorsal lateral geniculate nucleus (dLGN), these changes take place before the onset of patterned visually evoked activity [16]. However, it has been recently shown that DR can disrupt retinogeniculate connectivity by altering the synaptic strength and pruning of dLGN retinal connections [17, 18]. These changes prevail only when animals are put in the dark one week after eye opening and not when chronically deprived from birth. Interestingly, this form of dLGN visual experience-dependent plasticity occurs around the peak of visual cortical plasticity [15]. Whether CREB expression is upregulated in the dLGN, as it is in the visual cortex after DR, remains to be explored. To address this issue, we examined the effects of DR on CREB expression and phosphorylation in mouse dLGN using two DR protocols: chronic DR from birth (CDR) and DR following one week of vision (delayed DR, DDR).

## 2. Methods

### 2.1. Subjects

All procedures were performed in compliance with the Institutional Animal Care and Use Committee at Virginia Commonwealth University. C57BL/6 mice (Taconic Farms, Hudson, NY, USA) ranging in age from postnatal day (P) 0 to P27 were used in this study. The mice resided in colonies at the Virginia Commonwealth University Medical Center. Under normal light-rearing conditions (NR), mice were raised in a 12 hour light/12 hour dark cycle.

### 2.2. Dark Rearing (DR)

**Figure 1:**
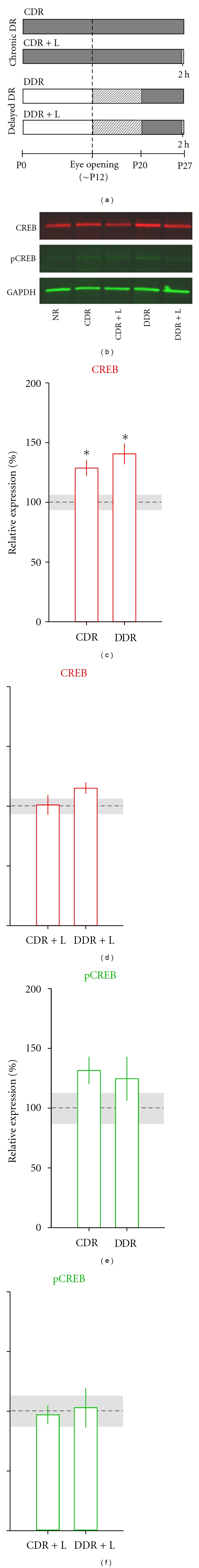
Dark rearing (DR) upregulates total CREB levels in the mouse dLGN. (a) Schematic showing the different DR conditions. The timeline depicts postnatal age in relation to the periods of DR (dark gray) and patterned visual experience (light gray). CDR and DDR; chronic and delayed DR; CDR + L and DDR + L; chronic and delayed DR followed by 2 hours of normal visual experience. Dotted line indicates the time of natural eye opening. For all conditions, tissue was harvested at postnatal day (P) 27. (b) Representative fluorescent immunoblot showing CREB (red) and pCREB (green) protein levels. GAPDH (green) was used as a loading control. CREB levels were higher in CDR and DDR compared to age-matched controls (normal-light reared, NR), and levels were restored after 2 hours of visual experience (CDR + L and DDR + L). pCREB levels were low and barely detectable (see the Results section for details). ((c)–(f)) Quantification of immunoblots (*n* = 5) for CREB ((c) and (d)) and pCREB ((e) and (f)) levels for DR ((c) and (e)) and DR + L conditions ((d) and (f)). The bars depict the relative averaged optical densities for each of the DR conditions (*n* = 6–8 dLGNs for each group per blot). Error bars represent ±SEM. The dotted line and corresponding shaded area reflect the averaged and ±SEM of CREB or pCREB protein levels obtained from age-matched controls. Total CREB levels for both DR conditions were significantly higher than control ((c), LSD post hoc test, *P < 0.05 for both conditions).

Dark-reared pups and their mothers were housed inside light-tight containers in a dark room. There were four DR conditions (Figure 1(a)): chronic DR, delayed DR, chronic DR + light, and delayed DR + light. For CDR, absolute light deprivation began at birth (P0) and lasted for 27 days. For DDR, animals were reared in normal light-dark cycle until P20 and then placed in the dark for 1 week. For CDR + L and DDR + L conditions, DR was followed by 2 hours of light exposure. All animals were sacrificed at P27. For CDR and DDR, mice were decapitated in complete darkness [19]. Daily care (changes of food, water, and litter) was performed in the dark using infrared viewing goggles.

### 2.3. dLGN Tissue Extraction and Sample Preparation

Mice were deeply anesthetized with isoflurane and decapitated. The brain was quickly removed and placed in a 4°C solution (in mM) of 2.5 KCl, 10 glucose, 126 NaCl, 1.25 NaH_2_PO_4_, 2 MgCl_2_, 2 CaCl_2_. 400 *μ*m thick sections were cut in the coronal plane with a vibratome (Leica VT1000S, Wetzlar, Germany). Tissue was extracted from sections through the middle of dLGN, then placed in a sucrose buffer (320 mM with 2% Complete Protease Inhibitor, Roche Diagnostics, Indianapolis, IN, USA) and stored at −80°C. Each sample contained the dLGN of both hemispheres from 3 to 4 animals. Whole tissue was homogenized in a sucrose buffer (320 mM with 2% Complete Protease Inhibitor and 1% PhosSTOP Phosphatase Inhibitor, Roche Diagnostics), and total protein concentration was determined using the Quick Start Bradford protein assay kit (Bio-Rad, Hercules, CA, USA). 

### 2.4. Western Blotting Using Odyssey Infrared Imaging

For gel electrophoresis, 20–30 *μ*g of protein for each sample was loaded onto a Criterion 10% Tris-HCl polyacrylamide gel (Bio-Rad). Then the protein was transferred to a nitrocellulose membrane (Bio-Rad). Membranes were blocked with the Odyssey blocking buffer (LI-COR, Lincoln, NE, USA). Rabbit anti-phospho-CREB (1 : 200; Cell Signaling, Danvers, MA, USA) and mouse anti-CREB (1 : 400; Cell Signaling) were diluted in Tris-buffered saline with Tween 20 (TBST) and 5% bovine serum albumin (BSA) and incubated overnight at 4°C. Rabbit anti-GAPDH (1 : 100,000; Cell Signaling) was used as a loading control. Then the membranes were treated with secondary antibodies conjugated to infrared (IR) fluorophores, IRDye 800CW goat anti-rabbit (1 : 10,000; LI-COR), and IRDye 680 goat anti-mouse (1 : 10,000; LI-COR), added to the blocking buffer for 1.5 hours at room temperature. 

### 2.5. Western Blotting Using Chemiluminescence Detection

Gel electrophoresis and transfer were performed as described above. Membranes were blocked with TBST and 5% powdered nonfat milk, then incubated with either rabbit anti-phospho-CREB (1 : 500; Cell Signaling) in TBST and 5% BSA or rabbit anti-CREB (1 : 500; Cell Signaling) in blocking buffer overnight at 4°C. Rabbit anti-GAPDH (1 : 100,000; Cell Signaling) was used as a loading control. The membranes were then treated with peroxidase goat anti-rabbit (1 : 2000; Jackson ImmunoResearch, West Grove, PA, USA), in blocking buffer for 1.5 hours at room temperature. SuperSignal West Pico Chemiluminescent Detection Reagent (Pierce, Rockford, IL, USA) was used to visualize immunoreactivity.

 Analysis for western blotting using IR imaging was performed using the Odyssey Application Software 3.0 (LI-COR). Analysis for western blotting using chemiluminescence detection was performed using Kodak 1D Image Analysis Software (Kodak, Rochester, NY, USA). Five separate measurements were taken for each band and the median value was used for estimating the protein levels. To minimize variability between blots, we assured that each blot had at least one sample from each group, so that relative changes to control could be examined within the same blot. Data were submitted to one-way analysis of variance (ANOVA) followed by post hoc comparisons to ascertain group differences. The least significant difference *t*-test (LSD) was used when equal variances were assumed and Tamhane's test when they were not. Significant differences between groups were defined as *P* < 0.05 (two tailed).

### 2.6. Immunofluorescence

Animals were perfused directly with 4% paraformaldehyde to preserve the phosphorylated state of CREB [8, 16]. Forty-micron-thick sections were then cut with a vibratome and blocked for 1 hour at room temperature using a solution of 2.5% BSA, 0.03% Triton X-100, and 5% normal goat serum in PBS. The primary antibodies were diluted in blocking solution, and sections were incubated in either rabbit anti-CREB (1 : 2000; Cell Signaling) or rabbit anti-phospho-CREB (1 : 800; Cell Signaling) overnight at 4°C. Sections were then incubated with secondary antibody (Alexa Fluor 488 goat anti-rabbit; 1 : 250; Invitrogen) in blocking solution for 2 hours at room temperature. Sections were placed on slides and mounted with ProLong Gold antifade reagent with DAPI (Invitrogen).

 To quantify cellular labeling of total CREB and phosphorylated CREB (pCREB), we measured the mean pixel intensity of fluorescence found in individual cells located within three to four 100 *μ*m × 100 *μ*m squares in dLGN. Measurements were conducted blind using Image J software and restricted to somata. Only those cells with a well-delineated continuous membrane within a given focal plane were included in the analysis. Using Metamorph software, a threshold was set so that there was a clear distinction between signal and residual background fluorescence [20]. Data were submitted to the Mann-Whitney tests to ascertain group differences. Significant differences between groups were defined as *P* < 0.05 (two tailed).

## 3. Results

We examined CREB expression in the dLGN of mice that were dark reared using western blot and immunofluorescence. Figure 1(a) provides a schematic delineating the different DR conditions. We first investigated the role of CREB in the dLGN of CDR (P0 to P27) and DDR (P20 to P27) P27 mice using infrared imaging detection. As shown in Figures 1(b) and 1(c), quantification of immunoblot optical densities indicated that both CDR and DDR led to a significant increase in total CREB levels (30–40%) relative to values obtained in age-matched controls (one-way ANOVA, *F* = 6.18, *P* < 0.05). However, CDR and DDR were not significantly different from each other (LSD post-hoc test, *P* = 0.25). These results are consistent with observations made in the mouse visual cortex indicating that DR per se leads to an increase in CREB-mediated transcription [10]. Interestingly, while our results show that both CDR and DDR lead to a comparable upregulation of CREB, DDR has a much greater impact on the retinogeniculate circuitry [17, 18].

We also investigated whether the upregulation of CREB observed after DR could be restored to control levels by returning animals to the light. Figures 1(b) and 1(d) show that 2 hours of light exposure brings CREB back to normal levels for both DR groups (CDR+L and DDR+L). These findings are in agreement with results showing that DR-induced synaptic disruptions in dLGN circuitry recover when animals were returned to a normally lit environment [18].

Quantification of immunoblot optical densities for pCREB protein levels revealed that, similar to total CREB, pCREB was upregulated after CDR and DDR and that these elevations returned to normal levels after 2 hours of light (CDR+L and DDR+L, Figures 1(b), 1(e), and 1(f)). However, the observed increase in pCREB levels after CDR and DDR was not significantly different from control (one-way ANOVA, *F* = 1.26, *P* = 0.32). The latter was likely due to the higher variability observed for pCREB optical density values (compare the ±SEMs of Figures 1(c) and 1(d) versus Figures 1(e) and 1(f)). Such increased variability could in part be explained by the fact that pCREB protein content is greatly reduced at these late postnatal ages [16] and represents only a fraction of the total CREB protein levels for all groups. Therefore, the strength of the optical signal for pCREB is relatively weak compared to background levels. Although always higher than background, averaged pCREB optical density measured across all conditions was only 3% (±0.52) higher than background levels. By contrast averaged total CREB was 21% (±2.04) higher than background.

**Figure 2:**
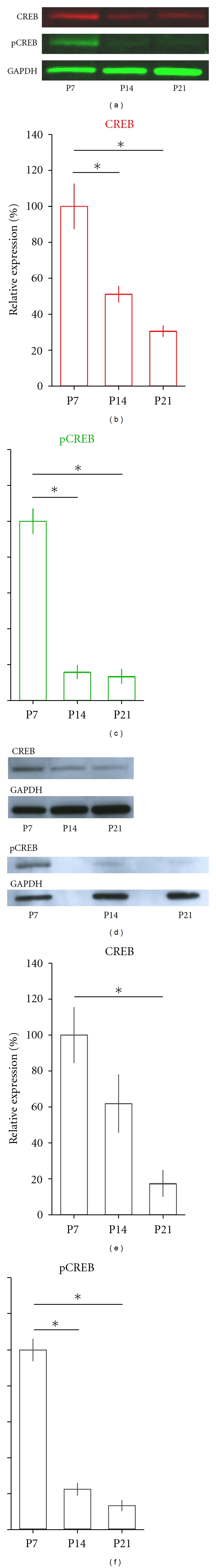
CREB and pCREB levels during early postnatal development in mouse dLGN. (a) Representative western blots of CREB and pCREB protein levels measured in dLGN at P7, P14, and P21 using infrared imaging detection. Red represents CREB levels and green pCREB and GAPDH levels. Both CREB and pCREB levels decreased with age. ((b) and (c)) Quantification of immunoblots for CREB (c) and pCREB (d) using infrared imaging. The bars depict the relative averaged optical densities measured at P7, P14, and P21. CREB and pCREB levels were significantly higher at P7 than at P14 and P21 (LSD post-hoc tests, *P < 0.01). (d) Representative western blots of CREB and pCREB protein levels measured in dLGN at the same postnatal ages as in (a) using chemiluminescence detection. ((e) and (f)) Quantification of immunoblots for CREB (e) and pCREB (f) using chemiluminescence. Similar to infrared imaging, CREB and pCREB levels significantly decreased with age ((e) LSD post-hoc test and (f) Tamhane's post-hoc test; *P < 0.01). Error bars represent ±SEM. Infrared imaging detection, *n* = 5 blots; chemiluminescence, *n* = 3-4 blots; *n* = 6–8 dLGNs in each age group per blot for both techniques.

To verify whether pCREB levels are downregulated early in development, we investigated CREB expression during the first postnatal weeks of the mouse dLGN (Figure 2). As shown in Figures 2(a) and 2(c), quantification of immunoblot optical densities confirmed that pCREB levels were high during the first postnatal week, then significantly decreased by P14 and continued to remain low at P21 (one-way ANOVA, *F* = 89.59, *P* < 0.001). A similar result was observed for total CREB levels (Figures 2(a) and 2(b); one-way ANOVA, *F* = 19.86, *P* < 0.001). In fact, similar results were obtained when the same experiment was carried out using chemiluminescence techniques (Figures 2(d)–2(f); one-way ANOVAs, *F* = 113.37 for pCREB, *F* = 9.18 for CREB, *P* < 0.05 for both cases). Thus, these findings indicate that CREB protein levels are developmentally regulated and greatly reduced after the third postnatal week. As a result, low protein levels may become problematic when averaging the relative optical densities of multiple blots and should be considered in future studies that make use of infrared imaging techniques.

**Figure 3:**
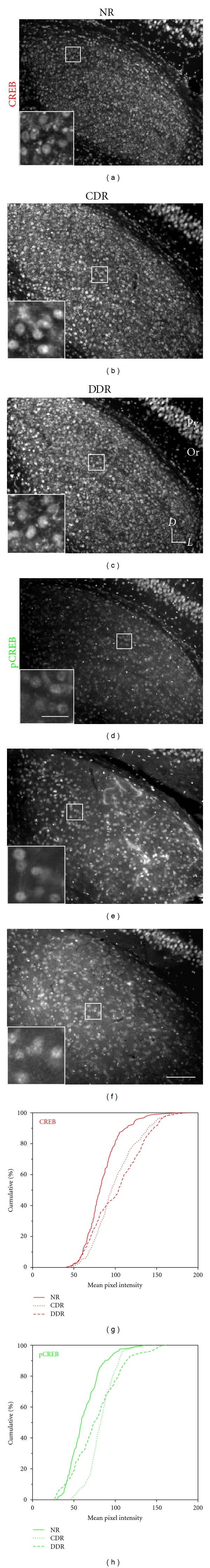
Modulation of CREB immunofluorescence in the dLGN of dark-reared mice. ((a)–(f)) Representative coronal sections of dLGN showing the labeling of total CREB ((a)–(c)) and pCREB ((d)–(f)), in NR ((a) and (d)), CDR ((b) and (e)), and DDR ((c) and (f)) mice. Corresponding high-magnification insets show that staining was prominent in somata. Py, pyramidal cell layer of hippocampus; Or, oriens layer of hippocampus; D, dorsal; L, lateral. Scale bars, 100 *μ*m and 25 *μ*m. ((g) and (h)) Cumulative histograms of cellular fluorescence intensity measured in somata in NR, CDR, and DDR for total CREB (g) and pCREB (h). Note that, for both total CREB and pCREB, CDR and DDR significantly shifted the distribution of fluorescence intensity toward larger values when compared to NR (the Mann-Whitney tests, *P* < 0.0001 for all comparisons). For CREB; NR *n* = 159 cells, CDR *n* = 267, DDR *n* = 224; for pCREB; NR *n* = 242 cells, CDR *n* = 276, DDR *n* = 144; from 2–6 dLGNs per group.

 To examine the pattern of neuronal CREB expression in CDR and DDR mice, we used immunofluorescence labeling techniques on fixed slices of dLGN (Figures 3(a)–3(f)). For both CREB and pCREB, cellular labeling was present throughout dLGN. High-power views of dLGN indicated that staining was highly enriched in somata (Figures 3(a)–3(f), insets). For CREB, labeling was also apparent in the surrounding neuropil (Figures 3(a)–3(c), insets). Interestingly, such extrasomatic staining seemed confined to dLGN and adjacent visual nuclei (e.g., intrageniculate leaflet and ventral lateral geniculate nucleus, not shown) but was absent from other nonvisual structures such as the hippocampus (Figures 3(a)–3(c)).

 Estimates of soma area (~100 *μ*m^2^) suggest that CREB and pCREB labeling was limited to relay neurons [20]. Similar to immunoblot results, both CREB and pCREB were upregulated after CDR and DDR. Interestingly, quantification of fluorescence signals within somata revealed significant increases in pixel intensity for DR groups compared to normal-light-reared (NR) animals (Figures 3(g) and 3(h)). This increase was true for total CREB levels (Kruskal-Wallis, *χ* ^2^ = 49.94, *P* < 0.0001) as well as for pCREB (Kruskal-Wallis, *χ* ^2^ = 126.65, *P* < 0.0001). Thus, our results are consistent with the proposal that DR upregulates the CREB pathway in response to decreased visual drive.

## 4. Discussion

Experiments in dark-reared mice suggest that normal-patterned vision is needed for the continued pruning of retinal connections onto dLGN cells as well as the synaptic strengthening of remaining ones [17, 18]. Here we show that a decrease in visual drive brought about by DR also upregulates total CREB protein levels in the dLGN. While we observed a concomitant increase in pCREB after DR, this change was not statistically different from protein levels obtained in controls. Because these measurements were obtained at late postnatal ages, a time when pCREB is extremely low compared to early postnatal ages, it could have made western blot quantification more difficult. Nonetheless, our immunofluorescence results indicate that both total CREB and pCREB are upregulated by DR. Thus, the changes in CREB reported here after DR suggests that this signaling pathway could contribute to plastic changes in retinogeniculate connectivity brought about decreased visual drive [17, 18]. Finally, it is important to note that, even though CREB expression was increased by DR, the degree of change was far below levels one sees at early postnatal ages, a time when retinal waves prevail and retinogeniculate projections are segregating into eye-specific domains [21].

Our results are consistent with observations made in visual cortex showing that DR upregulates the CREB transcription pathway [10]. However, in apparent contradiction to our findings, Pham et al. [8] showed that CRE-mediated transcription in dLGN is unaffected by visual deprivation. Perhaps this discrepancy is due to differences in deprivation paradigms, since Pham and colleagues [8] used monocular eyelid suture rather than DR. Indeed, monocular deprivation leads to unbalanced activity between the two eyes, whereas DR promotes an overall reduction of neuronal activity. In the developing visual cortex, these manipulations lead to different structural and functional outcomes [22–24] and also activate differentially a number of molecular pathways involved in activity-dependent plasticity, including CREB [10].

What are the possible mechanisms underlying the activation of the CREB pathway during DR? A common feature for many identified neuronal networks is the ability to adjust activity and cellular signaling in the face of decreased drive in an attempt to maintain an optimal balance between excitation and inhibition [25, 26]. It has been suggested that neurons are able to detect decreased firing rates through calcium-dependent mechanisms that regulate glutamate receptor trafficking [27]. Indeed, recent findings have implicated the CREB pathway in this process. For example, in mouse cultured cortical neurons, chronic activity deprivation activates cAMP signaling pathways, which in turn promote CREB-mediated transcription that subsequently leads to the synthesis of proteins necessary for AMPA receptor trafficking [28]. Interestingly in dLGN, decreased visual drive brought about by DR leads to a modification in glutamatergic synaptic transmission [17]. Whether the upregulation of CREB noted after DR is responsible for these changes or involves the activation of other molecular pathways in conjunction with CREB remains to be explored. For instance, manipulations that decrease neural activity affect a number of CREB-related signaling cascades including ones that involve brain-derived neurotrophic factor (BDNF) [29], the cytokine tumor necrosis factor *α* (TNF*α*) [30, 31], and the calcium/calmodulin-dependent protein kinase (CaMK) family [32–34].

It is important to note that CREB was upregulated whether DR was implemented from birth (CDR) or delayed (DDR) for one week or so after eye opening. Although both manipulations modify glutamatergic synaptic currents in dLGN, DDR appears to have a greater impact on retinogeniculate circuitry, weakening synaptic connections and altering the degree of retinal convergence [17, 18]. How then can we account for the fact that both CDR and DDR show comparable levels of CREB? One possibility is that the upregulation of CREB after DR reflects synaptic modifications other than those involving retinal inputs since dLGN receives afferent input from many sources, most notably excitatory feedback from the visual cortex [35, 36]. In fact, we have shown that corticogeniculate feedback can modulate the frequency and amplitude of miniature EPSCs recorded from dLGN cells after visual deprivation [37]. Another and perhaps more important possibility is that the *induction* of the reported modifications at the retinogeniculate synapse is driven by molecular pathways that are activated independently of CREB. However, our results suggest that, regardless of the molecular pathways underlying the induction of such changes, their *maintenance* seems to involve CREB. In fact, when visual activity is brought back to normal levels by exposing dark reared mice to the light, the upregulation of CREB is terminated.

In summary, our results reveal that the CREB pathway, while developmentally regulated in dLGN, can also be modulated by early visual experience. The latter extends the observations made in the visual cortex and suggests that the CREB pathway may represent a more generalized mechanism by which the developing visual system adapts to decreased visual drive.

## References

[1] Bailey CH, Bartsch D, Kandel ER (1996). Toward a molecular definition of long-term memory storage. *Proceedings of the National Academy of Sciences of the United States of America*.

[2] Silva AJ, Kogan JH, Frankland PW, Kida S (1998). CREB and memory. *Annual Review of Neuroscience*.

[3] Deisseroth K, Tsien RW (2002). Dynamic multiphosphorylation passwords for activity-dependent gene expression. *Neuron*.

[4] Frank DA, Greenberg ME (1994). CREB: a mediator of long-term memory from mollusks to mammals. *Cell*.

[5] Bito H, Deisseroth K, Tsien RW (1996). CREB phosphorylation and dephosphorylation: a Ca(2+)- and stimulus duration-dependent switch for hippocampal gene expression. *Cell*.

[6] Deisseroth K, Bito H, Tsien RW (1996). Signaling from synapse to nucleus: postsynaptic CREB phosphorylation during multiple forms of hippocampal synaptic plasticity. *Neuron*.

[7] Finkbeiner S, Tavazoie SF, Maloratsky A, Jacobs KM, Harris KM, Greenberg ME (1997). CREB: a major mediator of neuronal neurotrophin responses. *Neuron*.

[8] Pham TA, Impey S, Storm DR, Stryker MP (1999). CRE-mediated gene transcription in neocortical neuronal plasticity during the developmental critical period. *Neuron*.

[9] Mower AF, Liao DS, Nestler EJ, Neve RL, Ramoa AS (2002). cAMP/Ca2+ response element-binding protein function is essential for ocular dominance plasticity. *Journal of Neuroscience*.

[10] Tropea D, Kreiman G, Lyckman A (2006). Gene expression changes and molecular pathways mediating activity-dependent plasticity in visual cortex. *Nature Neuroscience*.

[11] Krahe TE, Wang W, Medina AE (2009). Phosphodiesterase inhibition increases CREB phosphorylation and restores orientation selectivity in a model of fetal alcohol spectrum disorders. *PLoS One*.

[12] Desai NS, Cudmore RH, Nelson SB, Turrigiano GG (2002). Critical periods for experience-dependent synaptic scaling in visual cortex. *Nature Neuroscience*.

[13] Wallace W, Bear MF (2004). A morphological correlate of synaptic scaling in visual cortex. *Journal of Neuroscience*.

[14] Kirkwood A, Rioult MG, Bear MF (1996). Experience-dependent modification of synaptic plasticity in visual cortex. *Nature*.

[15] Gordon JA, Stryker MP (1996). Experience-dependent plasticity of binocular responses in the primary visual cortex of the mouse. *Journal of Neuroscience*.

[16] Pham TA, Rubenstein JLR, Silva AJ, Storm DR, Stryker MP (2001). The CRE/CREB pathway is transiently expressed in thalamic circuit development and contributes to refinement of retinogeniculate axons. *Neuron*.

[17] Hooks BM, Chen C (2006). Distinct roles for spontaneous and visual activity in remodeling of the retinogeniculate synapse. *Neuron*.

[18] Hooks BM, Chen C (2008). Vision triggers an experience-dependent sensitive period at the retinogeniculate synapse. *Journal of Neuroscience*.

[19] Cancedda L, Putignano E, Impey S, Maffei L, Ratto GM, Pizzorusso T (2003). Patterned vision causes CRE-mediated gene expression in the visual cortex through PKA and ERK. *Journal of Neuroscience*.

[20] Jaubert-Miazza L, Green E, Lo FS, Bui K, Mills J, Guido W (2005). Structural and functional composition of the developing retinogeniculate pathway in the mouse. *Visual Neuroscience*.

[21] Guido W (2008). Refinement of the retinogeniculate pathway. *Journal of Physiology*.

[22] Fagiolini M, Pizzorusso T, Berardi N, Domenici L, Maffei L (1994). Functional postnatal development of the rat primary visual cortex and the role of visual experience: dark rearing and monocular deprivation. *Vision Research*.

[23] Katz LC, Shatz CJ (1996). Synaptic activity and the construction of cortical circuits. *Science*.

[24] Hensch TK (2005). Critical period plasticity in local cortical circuits. *Nature Reviews Neuroscience*.

[25] Turrigiano GG, Nelson SB (2004). Homeostatic plasticity in the developing nervous system. *Nature Reviews Neuroscience*.

[26] Maffei A, Nataraj K, Nelson SB, Turrigiano GG (2006). Potentiation of cortical inhibition by visual deprivation. *Nature*.

[27] Turrigiano GG (2008). The self-tuning neuron: synaptic scaling of excitatory synapses. *Cell*.

[28] Gong B, Wang H, Gu S, Heximer SP, Zhuo M (2007). Genetic evidence for the requirement of adenylyl cyclase 1 in synaptic scaling of forebrain cortical neurons. *European Journal of Neuroscience*.

[29] Rutherford LC, Nelson SB, Turrigiano GG (1998). BDNF has opposite effects on the quantal amplitude of pyramidal neuron and interneuron excitatory synapses. *Neuron*.

[30] Stellwagen D, Malenka RC (2006). Synaptic scaling mediated by glial TNF-*α*. *Nature*.

[31] Kaneko M, Stellwagen D, Malenka RC, Stryker MP (2008). Tumor necrosis factor-alpha mediates one component of competitive, experience-dependent plasticity in developing visual cortex. *Neuron*.

[32] Thiagarajan TC, Piedras-Renteria ES, Tsien RW (2002). *α*- and *β*CaMKII: inverse regulation by neuronal activity and opposing effects on synaptic strength. *Neuron*.

[33] Ibata K, Sun Q, Turrigiano GG (2008). Rapid synaptic scaling induced by changes in postsynaptic firing. *Neuron*.

[34] Doyle S, Pyndiah S, De Gois S, Erickson JD (2010). Excitation-transcription coupling via calcium/calmodulindependent protein kinase/ERK1/2 signaling mediates the coordinate induction of VGLUT2 and Narp triggered by a prolonged increase in glutamatergic synaptic activity. *Journal of Biological Chemistry*.

[35] Sherman SM (2007). The thalamus is more than just a relay. *Current Opinion in Neurobiology*.

[36] Bickford ME, Slusarczyk A, Dilger EK, Krahe TE, Kucuk C, Guido W (2010). Synaptic development of the mouse dorsal lateral geniculate nucleus. *Journal of Comparative Neurology*.

[37] Krahe TE, Guido W (2011). Homeostatic plasticity in the visual thalamus by monocular deprivation. *Journal of Neuroscience*.

